# The interaction of several herbal extracts with α-synuclein: Fibril formation and surface plasmon resonance analysis

**DOI:** 10.1371/journal.pone.0217801

**Published:** 2019-06-11

**Authors:** Shokouh Honarmand, Bahareh Dabirmanesh, Massoud Amanlou, Khosro Khajeh

**Affiliations:** 1 Department of Biochemistry, Faculty of Biological Sciences, Tarbiat Modares University,Tehran, Iran; 2 Department of Medicinal Chemistry, Faculty of Pharmacy and Drug Design and Development Research Center, Tehran University of Medical Sciences, Tehran, Iran; CSIR-Indian Institute of Chemical Biology, INDIA

## Abstract

Proteins from their native conformation convert into highly ordered fibrillar aggregation under particular conditions; that are described as amyloid fibrils. α-Synuclein (α-Syn) is a small natively unfolded protein that its fibrillation is the causative factor of Parkinson’s disease. One important approach in the development of therapeutic agents is the use of small molecules (such as flavonoids) that could specifically and efficiently inhibit the aggregation process. In this study the effect of few herbal extract (*Berberis*, *Quercus robur*, *Zizyphus vulgaris*, *Salix aegyptica*) containing flavonoids were investigated on fibril formation of α-syn by using conventional methods such as ThT fluorescence, circular dichroism (CD) spectroscopy and transmission electron microscopy (TEM).

The interaction of extracts were also analysed by surface plasmon resonance (SPR). Among extracts, *Salix aegyptica* revealed the highest inhibitory effect on fibril formation. As expected, *Salix aegyptica* extract also exhibited the highest affinity toward α-syn. Cell viability using MTT assay revealed that fibrils alone were more toxic than those containing the extract. Overall, we demonstrated that the affinity of compounds used in this study corresponds to their ability to arrest fibrillation and reduce cellular toxicity of α-syn fibrils.

## Introduction

The formation of amyloid is one of key characteristics in many neurodegenerative diseases such as Alzheimer’s, Parkinson’s and Huntington’s diseases. In these diseases, under particular conditions, structure of proteins undergo gradual conformational conversion to β-rich amyloid fibrils [1, 2]. Parkinson’s disease is the second common neurodegenerative disorder after Alzheimer’s which is characterized by filamentous α-synuclein within Lewy bodies followed by loss of dopaminergic neurons in the substantia nigra [3, 4]. α-synuclein (α-syn) is considered as an extremely heat-resistant, small acidic protein (14 kDa) which have little or no ordered secondary structure under physiological condition [5–8]. Its monomers at high concentration and under oxidative stress in the presence of metal ion are prone to form β-sheet-rich oligomer species and eventually stable fibrils. These proteinaceous deposits cause neurodegeneration and the onset of Parkinson symptoms [9]. So, inhibiting the amyloid fibril growth has been considered as one therapeutic approach for this disease [10]. Recent studies have identified that flavonoids which belong to a class of polyphenols have anti-amyloidogenic properties against protein misfolding and amyloid formation. Flavonoids are secondary metabolites of plants found abundantly in a variety of herbal extracts, fruits, vegetables and beverages [11–13]. Small molecules that are capable of binding to α-syn could probably inhibit fibrillation; therefore, using surface plasmon resonance (SPR) with the ability to study molecular interactions between a wide variety of label free molecules could be a useful technique to screen fibril formation inhibitors. To date, several methods such as TEM, Congo red binding, Thioflavin-T assay, light scattering and atomic force microscopy (AFM) have been used to study amyloid fibril formation [14]. In the current study, several herbal extracts (*Berberis*, *Quercus robur*, *Stigma maydis*, *Salix aegyptica*) were first chosen and their anti-fibrillation effect on α-syn aggregation were first investigated using conventional methods such as ThT assay, TEM and CD spectroscopy. Then their interaction with α-syn was evaluated using surface plasmon resonance (SPR). The results of this study could be helpful in designing a new and effective SPR based approach for screening anti-amyloidogenic compounds.

## Materials & methods

### Materials

All chemicals were purchased from Sigma–Aldrich Chemical (USA). The carboxymethyldextran (CMD 500 D) sensor chip, N-hydroxysuccinimide(NHS), N-ethyl-N-(3-diethylaminopropyl) carbodiimide (EDC), and ethanolamine hydrochloride were obtained from Xantec Bio-analytics (Germany). All the reagents used in this study were of analytical grade.

### Expression and purification of α-syn

α-syn was expressed in *E*.*coli* BL21 (DE3). The cells were cultured at 37°C and180 rpm in LB medium containing kanamycin (50 μg/mL). After reaching 0.5 at 600 nm (OD600), expression was induced with 1mM IPTG for 4 h. The cells were harvested, suspended in lysis buffer (40 mM Tris, 5 mM imidazole, 400 mM NaCl) and disrupted using sonication. Disrupted cells were removed by centrifugation (1800 xg, for 20 min at 4°C). To obtain purified protein, the supernatant was loaded on Ni-agarose column. α-syn was eluted at 100 mM imidazole. The purity of elution fractions was confirmed by %12 SDS-PAGE [15].

### Herbal extraction

Powdered seeds of *Berberis*, *Quercus robur*, *Stigma maydis* and *Salix aegyptica* were extracted in 2000 mL methanol/water (80:20,v/v) for 24 h at room temperature (25 ºC), followed by 2 h sonication in ultrasonic bath. Then the extracts were concentrated by rotary evaporator and kept in -20 ºC.

### α-syn fibril formation

Purified protein from previous stage was dialyzed by a 3-kDa dialysis membrane against 30 mM Tris and 200 mM NaCl buffer, pH 7.5. To gain a suitable concentration for fibrillation, protein was concentrated using centricon (MWCO 3kDa). Protein concentration was quantified using absorbance at 280 nm (ε = 5960 cm^-1^ M^-1^) [16]. Final concentration of α-syn was 2.2 mg/mL.

Fibril formation was carried out in a mixture (final volume of 525 μL) containing 500 μL of α-syn (2.2 mg/mL) in 50 mM Tris buffer (pH 7.5) and 200 mM NaCl at 37°C while stirred using micro stir-bars. To analyse the effect of extracts on α-syn fibrillation 25 μl of each extract (1 mg/mL) was added into 500 μL of protein solution and incubated under noted condition. Fibril formation was monitored with fluorescence using 10 μL of each sample at regular time intervals and mixed with 1000 μL of 25 μM ThT.

### ThT assay

A 1 mM aqueous solution of Thioflavin T was prepared in 25 mM phosphate buffer (pH 6) and filtered through 0.2 μm polyether sulfone filter. Subsequently, aliquots (10 μL) were taken out at regular time intervals from each incubated samples and mixed with 1000 μL of ThT (25 μM) to monitor fibril formation by ThT assay. The molar ratio of final protein concentration and ThT concentration was about 1 to 15.The samples were excited at 440 nm and the resulting ThT fluorescence spectra of samples were measured between 450–520 nm using LS55 PerkinElmer fluorescence spectrometer. The excitation and emission slit were both set to 5 nm. The plots of ThT fluorescence at 485 nm versus time were obtained. Percent changes in fluorescent intensity was calculated using Eq 1.

%Fluorescenceintensity(FI)=[Fe−Fs]/Fs×100 Eq 1

Fe and Fs were intensities obtained from α-syn fibrils formed in the presence and absence of extract, respectively. A reduction in the amyloid fibril formation was associated with a decrease in the emission intensity [16].

The percent inhibition was determined using changes in the intensity in the presence and absence of each extract (Eq 2) [16].

(%)inhibition=(percentFIinthepresenceofextract/percentFIforα‐synalone)×100‐100 Eq 2

### Transmission electron microscopy (TEM)

20 *μ*L drop of the sample was placed on a Carbon film coated on 300 mesh copper grid (Agar) for 2 min. Excess liquid was absorbed with filter paper then negatively stained with a 20 *μ*L drop of 2%

uranyl acetate for 1–2 min and excess liquid was absorbed with filter paper. The grid was allowed to dry and examined on a Zeiss EM10C transmission electron microscope operating at an accelerating voltage of 100 kV.

### CD spectroscopy

Far UV spectra were conducted using a JASCO J-715 spectropolarimeter (Japan). The CD spectra were taken at protein concentration of 0.25 mg/mL in 30 mM Tris buffer (pH 7.5) containing 200 mM NaCl using 1 mm path length cell. All spectra were collected from 200 to 245 nm and were corrected by subtracting a blank spectrum. Noise in the data was smoothed using the JASCO J-715 software. Results are expressed as molar ellipticity, (θ) deg cm^2^dmol^−1^, that was calculated from the formula [θ] λ = [θ × 100 MRW]/(cl), were c is the protein concentration in mg/mL, l the light path length in centimetres, and θ; the measured ellipticity in degrees at wavelength λ.

### SPR procedures

Biomolecular interactions of α-syn with Herbal extracts were investigated using Xantec SR7500DC instrument (Germany), equipped with an automatic flow injection system. The instrument detects changes in the refractive index as micro- refractive-index (mRIU), which is proportional to the quantity (mass) of analyte interacting with the surface. CMD500 chip was used in this study. The immobilization of α-syn on the CMD chip surface was performed based on amine coupling method. The activation of the surface carboxyl group was carried out by injecting of EDC/NHS for 10 min. Then 250 μl of α-syn (0.2 mg/mL) in 10 mM acetate buffer (pH 4.5) was injected over the activated chip. Subsequently, the remaining activate sites of the chip were blocked using 1 mM ethanolamine (pH 8.5). All steps were followed by rinsing with a phosphate buffer solution. After α-syn immobilization, 50 ng/mL of each extract solutions (pH 5.5) were injected over the chip surface for 2.5 min with a flow rate of 100 μL/min at 25°C. After each analysis cycle, regeneration buffer containing 1 M NaCl was injected to remove bounded analyte. Finally, reference sensorgram was subtracted from binding sensorgrams using the Scrubber analysis program (Biologic Software Pty. Ltd., Canberra, Australia).

### MTT assay

The viability of cells was analysed using MTT assay that determines the mitochondrial dehydrogenase activity. The human neuroblastoma cell line SH-SY5Y was obtained from Pasteur institute, Iran. Cells were grown at 37 ºC in an incubator with 5% CO2 in Dulbecco’s modified Eagle medium (DMEM, GIBCO) supplemented with 10% fetal bovine serum (FBS, GIBCO). Subsequently, cytotoxicity was evaluated using colorimetric MTT [3-(4, 5-dimethyl-2-thiazolyl)- 2, 5-diphenyl-2Htetrazolium bromide] assay [17]. SH-SY5Y cells were seeded at 20000 per well in 96-well plates. After 36 h, the medium was removed and replaced with media containing extract, oligomers formed from α-syn alone and oligomers from α-syn co-incubated with extract. Untreated cells (cells + medium) were used as a control. All samples were then left at 37 ºC. After 24 h, medium was replaced with MTT (5 mg/mL) in PBS. The cells were incubated for another 4 h. Then MTT was removed and the formazan crystals were dissolved in DMSO (produced purple colour). The amount of formazan was analysed by measuring the optical density at 570 nm. Cell viability was expressed as the percentage relative to the control cells. Experiments were performed at least in triplicate and the standard deviations were below ±5%.

## Results & discussion

### Expression & purification of α-syn

To analyse the effect of extracts on fibrillation, α-syn was first expressed and then purified. As impurities have an increasing effect on the process of fibrillation [18], the purity of α-syn was confirmed by SDS–PAGE analysis (S1 Fig). α-syn has a molecular mass of 14 kDa but it migrates on SDS-PAGE with the apparent mass of ~ 20 kDa due to its poor binding to SDS [19].

Since kinetics of fibrillation is based on a nucleation-dependent mechanism, therefore, the influence of environmental variables (such as protein and NaCl concentration) could affect the pathway of assembly, and the final fibril morphology [20]. In this study, the concentration used for α-syn fibril formation was 2.2 mg/mL in the presence of 200 mM NaCl (S2 Fig).

### Effect of extracts on α-synuclein fibrillation

The effect of different extracts on the fibril formation of α-syn was determined by ThT fluorescence, the most common long-established method to detect, diagnose, and analyse of amyloid fibrils [21].

**Fig 1:**
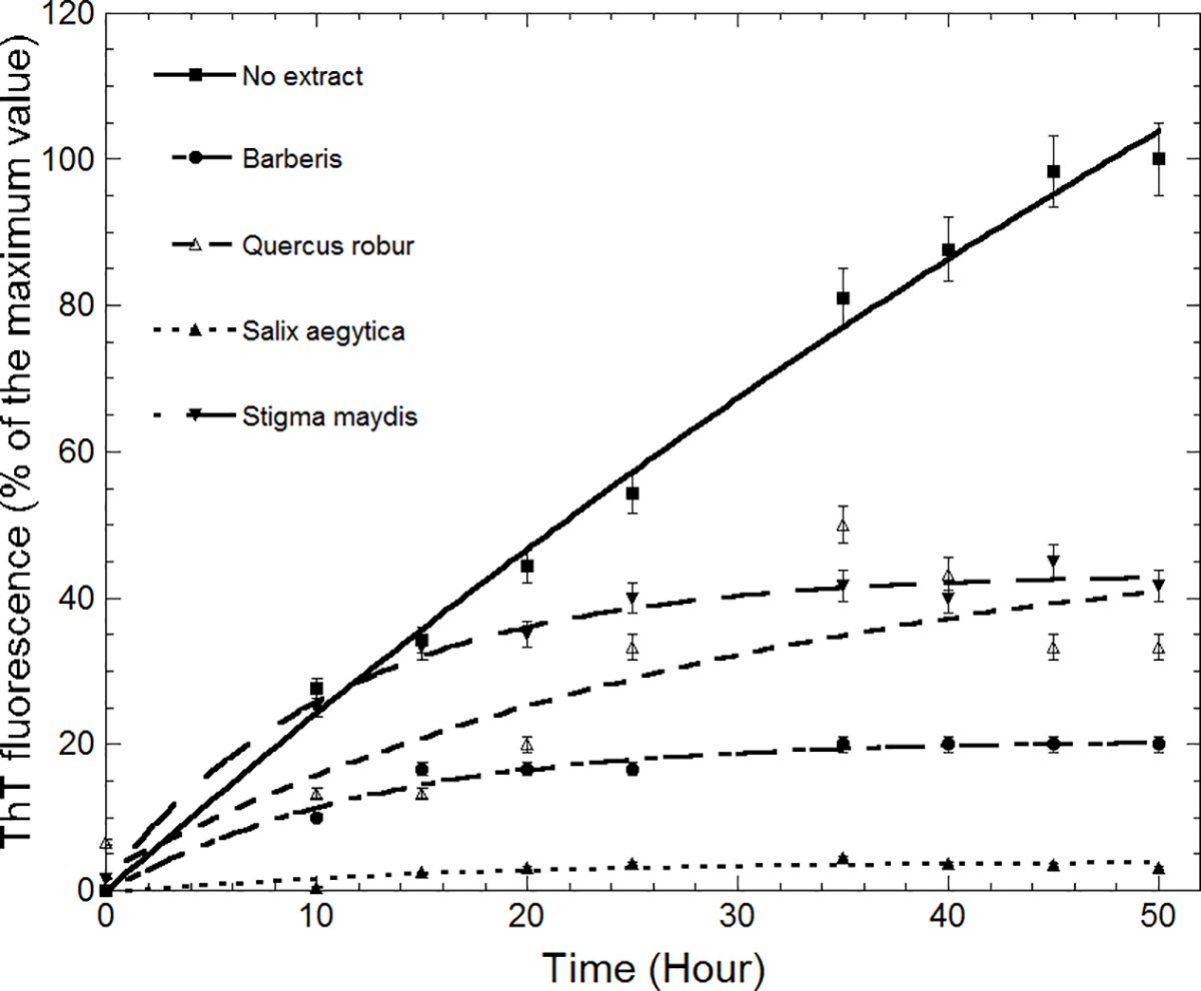
Kinetics of α-syn fibrillation. Time course of α-syn fibrillation in the presence and absence of extracts. α-syn was co-incubated with 50 μg/mL of each extract. The ability of these extracts to prevent aggregation is shown as percentage increase in ThT fluorescence intensity. At various time intervals, 10 μL of aggregated samples were taken and incubated with 1mL of ThT for 15 minutes at room temperature. Then, samples were excited at 440 nm and their emissions recorded at 480 nm. A positive control contained α-syn alone in Tris buffer (pH 7.5) (solid line) and a negative control was α-amylase with no intensity at given wavelengths. Results are the mean of three different experiments and the standard deviations are below ±5%.

**Fig 2:**
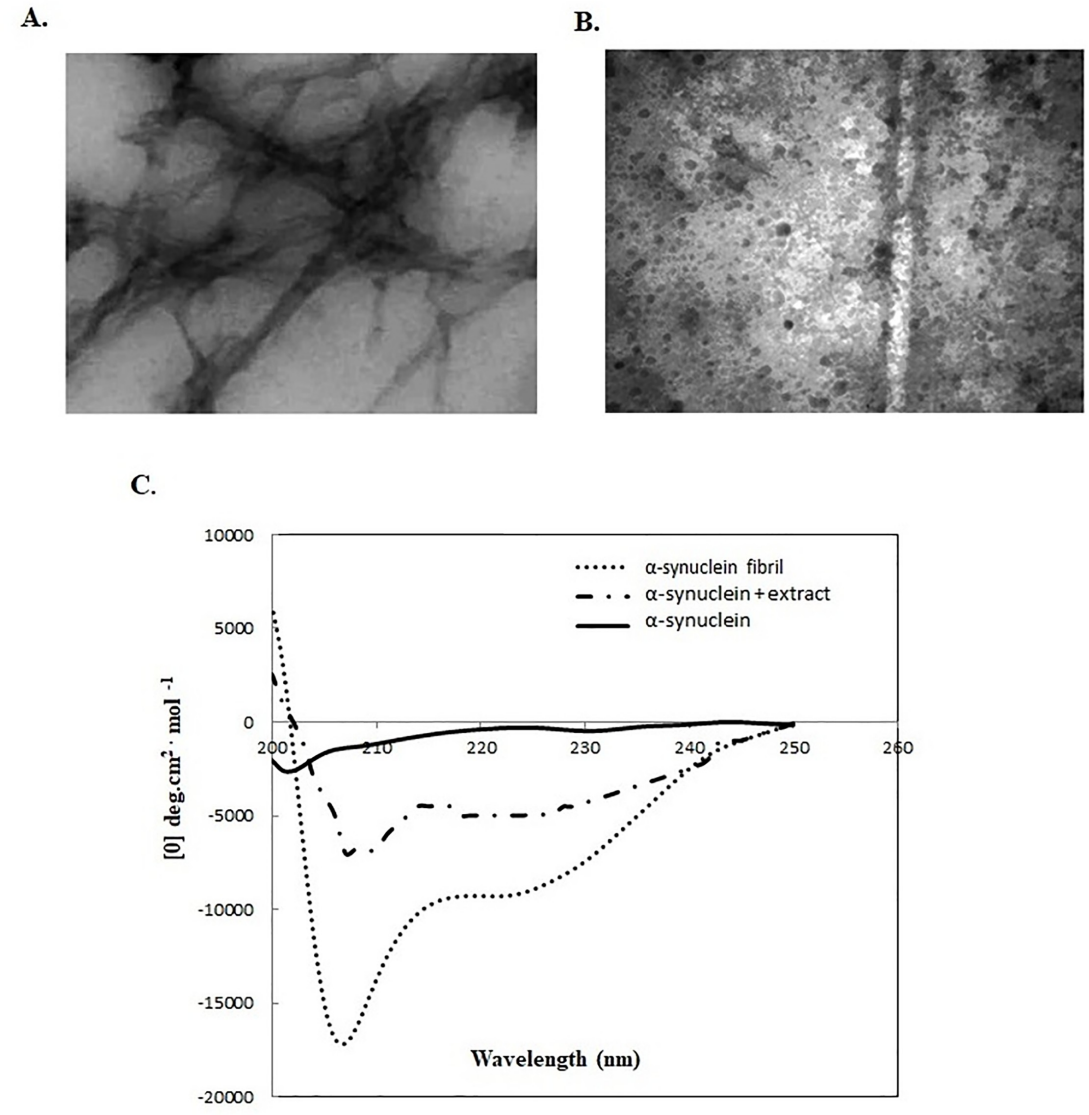
TEM images and CD spectroscopy of fibrils. (A) TEM images of fibrils formed in the absence and (B) presence of *Salix aegyptiaca*. (C) CD spectra of α-syn in the far-UV region showing secondary structural changes as a fibril formation in the presence and absence of *Salix aegyptiaca*.

ThT is a benzothiazole dye that as it binds to amyloid fibrils (ordered beta sheet-rich structures) exhibits an intensified fluorescence whereas unbound ThT is not fluorescent at this wavelength [21, 22]. Therefore, ThT is mostly used as an extrinsic probe for monitoring fibrillization kinetics in vitro and in the presence of fibrils gives rise to an emission at 480 nm due to an increase in beta sheet structures. To compare the effects of different extracts on fibril formation, ThT fluorescence was plotted as a function of time. As shown in Fig 1, when α-syn co-incubated with extracts the fluorescence emission of ThT declined remarkably indicating a lower fibril formation. The fluorescence intensity of α-syn fibrils in the presence and absence of each extract was compared and their amyloid fibril inhibitory effect was determined. Among the extracts, *Salix aegyptiaca* had the highest inhibitory effect on α-syn fibrillation (up to 95%) when compared to the control. *Quercus robur* and *Stigma maydis* had similar inhibitory effect (60%) however; they were less potent than *Berberis* (80% fibril formation inhibition). Previously *Stigma maydis* was reported as a valuable source of bioactive components with inhibitory effect of amyloid β aggregation [23]. To verify the results from ThT assay and provide visual proof, TEM images obtained. The morphology of aggregates produced from α-syn alone and α-syn co-incubated with the most effective extract (*Salix aegyptiaca*) was compared. TEM images of α-syn fibrils formed in the absence of extract revealed a detectable thin, long fibrils in clusters. Our results are similar to the previous TEM images obtained for α-syn fibrils [16]. However, the amount of fibrils formed has considerably decreased and a very small amount of small oligomers was observed in presence of *Salix aegyptiaca* (Fig 2A & 2B). Based on previous studies herbal extracts are composed of combination of various unknown chemicals and the reason for their anti-amyloidogenic effect could not be discussed precisely. However, the presence of flavonoids and phenolic compounds in *Salix aegyptiaca* extract were previously verified [24–27]. Polyphenols were reported to inhibit fibril formation by maintaining the native structure of α-syn and preventing the formation of any intermediate molten globular or β-sheet conformation that are essential for amyloid formation [11, 28–30]. In order to characterize the secondary structure and detect the presence of β-sheet structure of the aggregates far-UV CD spectroscopy was used. The far-UV CD spectra of α-syn alone at time 0 h and at time 50 h were acquired. As shown in Fig 2C the far-UV CD spectrum of α-syn at time 0 is flat at wavelengths from 210 to 250 nm and has a negative peak between 195–200 nm, implying unfolded protein. On the other hand, the CD spectrum of sample incubated at 37°C, under fibrillation condition, displayed a negative peak centred at 217 nm, indicating the presence of β-sheet structure (Fig 2C). However, CD spectrum (Fig 2C) of α-syn fibrils formed in *Salix aegyptiaca* exhibited a decrease in β-sheet secondary structure when compared to the control (without extract) that could be due to the reduction in fibril formation. High amounts of phenolic compounds including gallic acid, caffeic acid, vanillin, р-coumaricacid, myricetin, catechin, epigallocatechin gallate and flavonoids such as rutin, quercetin and salicin have been reported in *S*. *aegyptiaca* extract [24, 25]. Previously reported that flavonoids, such as quercetin could significantly inhibit α-syn fibrillation [29–32]. The importance of the galloyl moiety on anti-amyloidogenic activity has also been reported previously [11, 29, 32]. Most flavonoids proved effective against abnormal misfolding due to their aromatic rings essential for π–π stacking interactions with hydrophobic amino acid residues and their hydroxyl groups that form hydrogen bonds with hydrophilic amino acid residues [30]. Stochastic conformational analysis *in silico* performed by Kuzuhara and colleagues revealed many conformations of epicatechin gallate indicating that the mobility and flexibility of the galloyl moiety allow these compounds to take on multiple conformations that may be relevant for interaction with different molecular targets [30].

**Fig 3:**
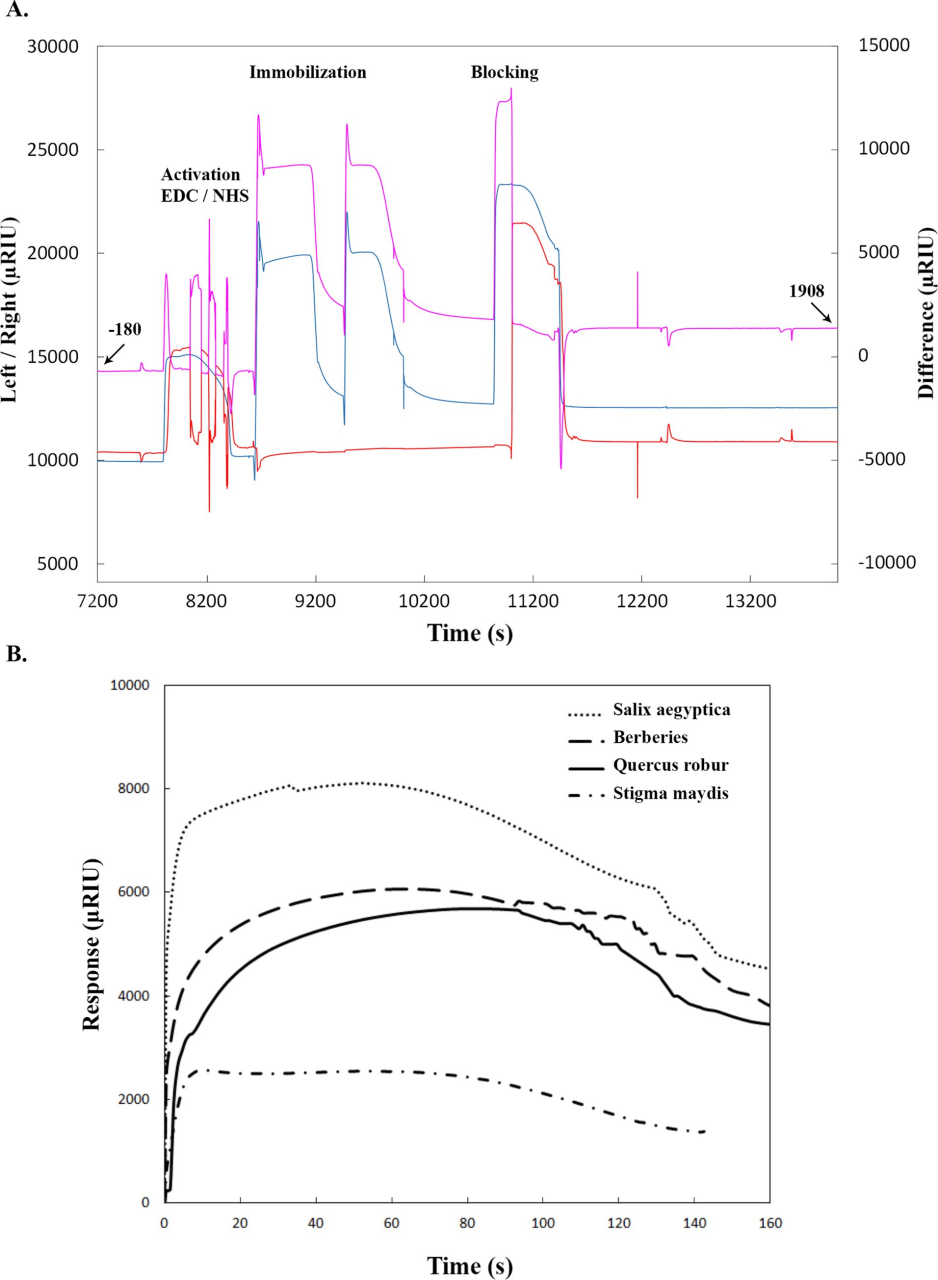
SPR analysis for extracts affinity toward α-syn. (A) Sensogram for covalent immobilization of α- synuclein onto SPR-CMD500 D surface. Running buffer: 20 mM phosphate buffer (pH 7.5), flow speed 25 μ l/min. Immobilization buffer: 10 mM sodium acetate buffer (pH 4.5) for 10 min. Blocking: 1M ethanolamine (pH 8.5). The solid lines representation; blue line: Sample side, red line: Reference and pink line: Difference. (B) Sensorgrams for the interaction of extracts with immobilized α-syn on CMD500D. Extract mixtures (in phosphate buffer pH 5.5) were injected over the chip surface for 2.5 min with a flow rate of 100 μl/min at 25°C.

Assuming that compounds with higher affinity toward α-syn could serve as a more effective fibril formation inhibitors encouraged us to investigate the interaction of each extract with α-syn. Previously, Sun *et al*. reported the specific binding of α-syn oligomer to aptamer using SPR [33]. In addition, Kang *et al*. used SPR response to monitor the urea-induced denaturation of surface bound α-syn [34]. Furthermore, among the techniques available to determine binding affinities, surface plasmon resonance has emerged as the gold standard and widely used optical detection technique for measuring the strength and rate of biomolecule interactions with high sensivity, low detection limit and less sample consumption [33–35]. This approach could be used for screening amyloid inhibitors without using extrinsic dye and eliminate optical artifacts such as absorbance overlap by ligands and light scattering due to fibril formation. For these reasons, we decided to develop a direct assay to determine the interaction of the extracts toward α-syn by surface plasmon resonance measurement. As mentioned earlier, α-syn belongs to natively unfolded proteins, which has no distinguished 3D structures under physiological condition. This protein consists of 15 lysine residues [5], therefore, immobilization of α-syn on the CMD500D chip surface was carried out via lysine residues using amine coupling method. Pre-concentration was performed to find the appropriate immobilization pH. The optimum pH value was found to be 4.5. Subsequently, carboxyl groups of CMD500D chip surface were activated with a mixture of EDC and NHS to give reactive succinimide esters. α-syn was then passed over the surface in 10 mM sodium acetate buffer (pH 4.5) and linked to the sensor chip via amine coupling. Unreacted NHS esters were then blocked with ethanolamine. An increase in the SPR signal up to 2000 μRIU was achieved by protein immobilization (Fig 3A). For the reference flow cell, only activation and blocking was carried out in the same way as mentioned above. After covalent immobilization on carboxymethyl dextran matrix, each herbal extract was dissolved in Tris buffer (30 mM Tris, 200 mM NaCl with %5 DMSO, pH 4.5) and injected simultaneously over the immobilized α-syn and reference channel. Passing the extracts over the chip surface were accompanied by an increasing in SPR signal. SPR signal increased upon injection of plant extracts into sensor chip and when the ligand-α-syn binding process reached equilibrium, running buffer was passed over the sensor and dissociation of ligand was occurred. Eventually, SPR signals returned to the baseline, and showed that the plant extracts have been reversible bonded to α-syn. The highest SPR response was observed for *Salix aegyptica* while the least was for *stigma maydis* (Fig 3B). The SPR response is directly related to the mass changes on the sensor surface due to higher amount of extract attached to α-syn. So, the SPR response indicates the strength of interaction between analyte and ligand. Among selected herbal extracts, *Salix aegyptica* with the SPR response of 8000 μRIU interacted strongly with immobilized α-syn and *stigma maydis* extract with the response of 2000 μRIU had the weakest interaction. Both *Berberis* and *Quercus robur* exhibited the response of 5000 μRIU. Results obtained from SPR assay were in good agreement with other results obtained in this study, suggesting that extracts with higher affinity with α-syn could have more inhibitory effect on fibrillation.

### Cytotoxicity

Any method to find new anti-amyloidogenic molecules should take into account the toxicity of the resulting oligomers. Recent studies have shown that extracellular α-syn fibrils can be transported to neurones and be mostly responsible for α-syn pathology. Also obtaining a relationship between the affinity and cytotoxicity could be beneficial.

**Fig 4:**
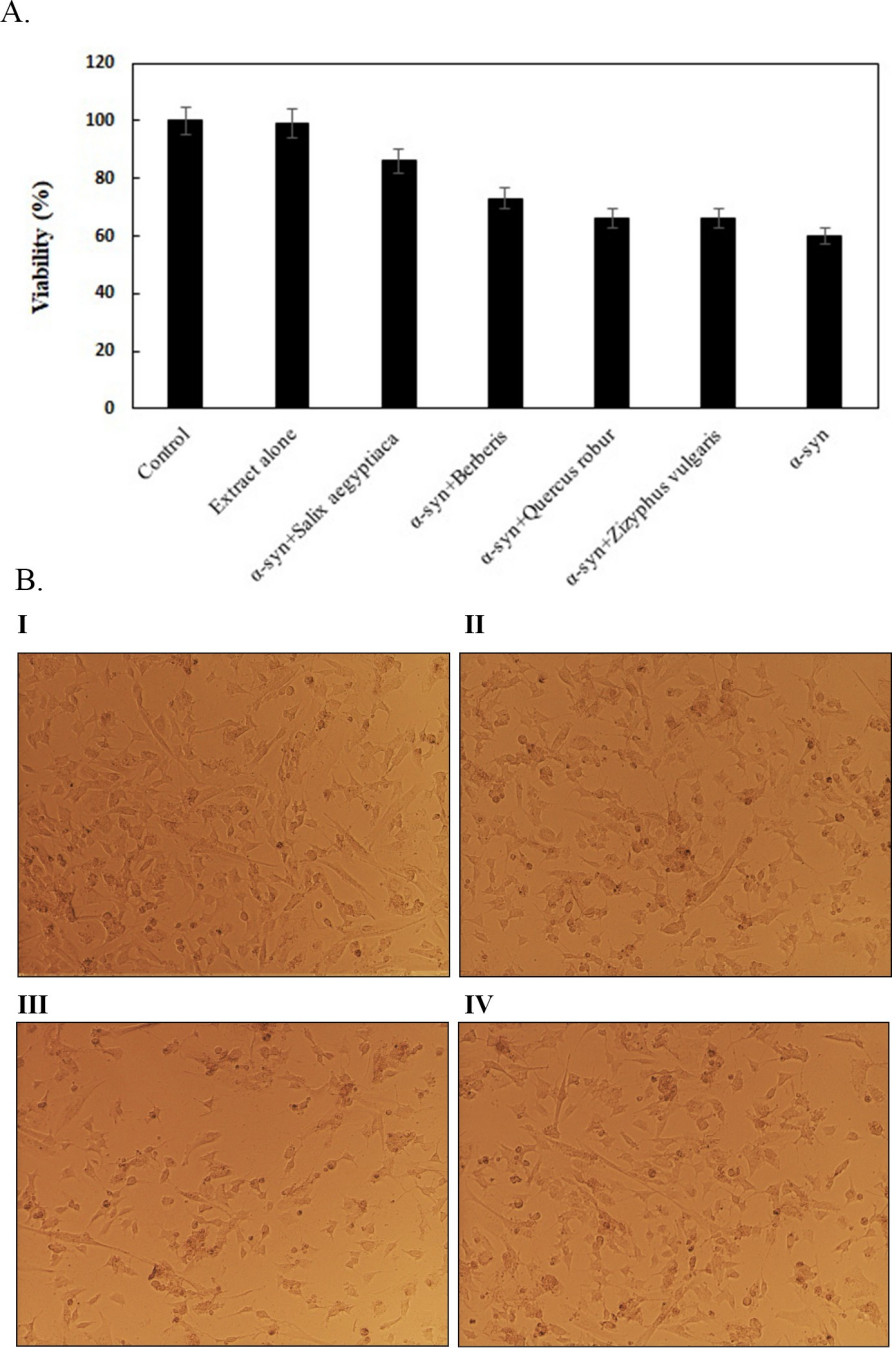
Cell viability. (A) MTT assay for oligomers formed from α-syn alone and α-syn co-incubated with extracts. α-syn fibrillation was carried out in the presence and absence of each extract. SH-SY5Y cells were co-incubated with the fibrils formed from above samples for 24 h followed by the addition of MTT. The products of MTT reduction was a purple colour that was read at 570 nm. The data were finally expressed as a percentage relative to the control (cells without treatment). The cell viability of the control sample was set as 100% viable. (B) Appearance of SH-SY5Y cells under microscope (Nikon Eclipse TE2000-S) after 24h incubation. The presence of the extract during fibrillation reduced cytotoxicity due to lower fibril formation. I: cells without extract, II: cells treated with extract alone, III: cells treated with α-syn fibrils and IV: cells treated with α-syn fibrils formed in the presence of *Salix aegyptiaca* extract.

Therefore, SH-SY5Y cells were contaminated with extracellular α-syn fibrils formed in the presence and absence of mentioned extracts, similar to the pathophysiological situation. α-syn fibrils formed in the absence of extracts exhibited the highest cytotoxicity (60% viability). However, cells incubated with fibrils formed in the presence of *Salix aegyptiaca* and *Berberis* extracts demonstrated 86% and 72% viability, respectively (Fig 4A). As it was expected, in the presence of extracts toxicity was reduced when compared to the α-syn fibrils alone due to lower fibril formation. Extracts with highest affinity towards α-syn exhibited lower fibril formation and toxicity. Previously, other researchers also reported the similar results on the effect of natural products on the reduction of α-syn fibrillation and cytotoxicity [16, 36]. The oligomers of α-syn formed with incubation of *Salix aegyptica* extract were not toxic by nature. *Salix aegyptiaca* with highest affinity toward α-syn showed the highest viability (Fig 4A & 4B). Similar to our results, Cairo et al. reported that ligands exhibiting greater affinity for β-amyloid are more effective for inhibiting aggregation and cell toxicity [37].

## Conclusion

A large group of diseases are associated with the formation of amyloid fibril. A common therapeutic strategy is to develop protein aggregation inhibitors that can slow down or prevent amyloid formation. One strategy is to screen for small molecules by assessing their binding affinity to their target protein. Since SPR assay is convenient, reproducible, and has the necessary sensitivity to detect the interaction of low molecular weight ligands, we developed a SPR-based analytical system for screening anti fibrillation compounds. Using our direct assay in combination with conventional methods (ThT fluorescence, TEM and CD spectroscopy) revealed that the extract (*Salix aegyptiaca*) with highest affinity toward α-syn could exhibit the highest anti amyloidogenic effect and could be used as a potential anti-fibrillation therapeutic drug which require further investigation.

## Supporting information

S1 Fig SDS-PAGE for purified α-syn by NiNTA column. (TIF)

S2 Fig α-syn fibrillation. (A)The effect of different concentration of protein on fibril formation of α-synuclein in 50 mM Tris buffer pH 7.5 at 37 ºC. (B) The effect of NaCl concentration on the α-synuclein (2.2 mg/mL) fibrillation in Tris buffer pH 7.5 at 37 ºC with constant agitation using a small magnetic stirring bar.
(TIF)
