# Supplementary figures and images for: The interaction of several herbal extracts with α-synuclein: Fibril formation and surface plasmon resonance analysis
Source: PLoS One. 2019 Jun 11;14(6):e0217801. doi: 10.1371/journal.pone.0217801 (PMC6559707; doi:10.1371/journal.pone.0217801)

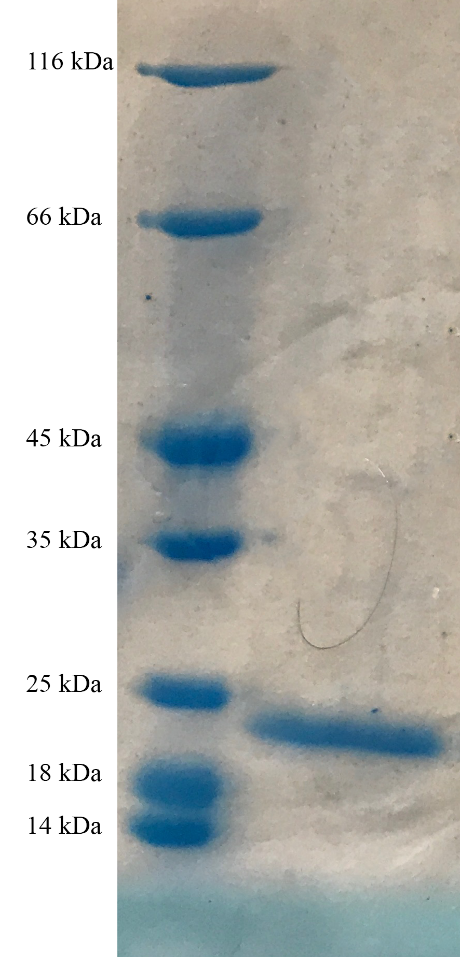

Supplement: S1 Fig — (TIF) [file pone.0217801.s001.tif]

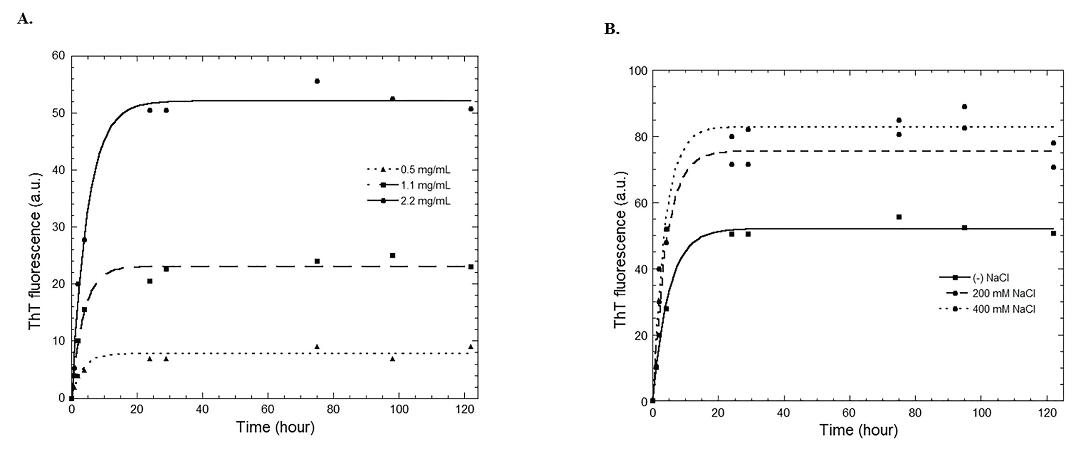

Supplement: S2 Fig — (A)The effect of different concentration of protein on fibril formation of α-synuclein in 50 mM Tris buffer pH 7.5 at 37 ºC. (B) The effect of NaCl concentration on the α-synuclein (2.2 mg/mL) fibrillation in Tris buffer pH 7.5 at 37 ºC with constant agitation using a small magnetic stirring bar. (TIF) [file pone.0217801.s002.tif]
